# Fitness and Phenotypic Characterization of Miltefosine-Resistant *Leishmania major*

**DOI:** 10.1371/journal.pntd.0003948

**Published:** 2015-07-31

**Authors:** Kimbra G. Turner, Paola Vacchina, Maricela Robles-Murguia, Mariha Wadsworth, Mary Ann McDowell, Miguel A. Morales

**Affiliations:** Eck Institute for Global Health, Department of Biological Sciences, University of Notre Dame, Notre Dame, Indiana, United States of America; University of Antwerp, BELGIUM

## Abstract

Trypanosomatid parasites of the genus *Leishmania* are the causative agents of leishmaniasis, a neglected tropical disease with several clinical manifestations. *Leishmania major* is the causative agent of cutaneous leishmaniasis (CL), which is largely characterized by ulcerative lesions appearing on the skin. Current treatments of leishmaniasis include pentavalent antimonials and amphotericin B, however, the toxic side effects of these drugs and difficulty with distribution makes these options less than ideal. Miltefosine (MIL) is the first oral treatment available for leishmaniasis. Originally developed for cancer chemotherapy, the mechanism of action of MIL in *Leishmania* spp. is largely unknown. While treatment with MIL has proven effective, higher tolerance to the drug has been observed, and resistance is easily developed in an *in vitro* environment. Utilizing stepwise selection we generated MIL-resistant cultures of *L*. *major* and characterized the fitness of MIL-resistant *L*. *major*. Resistant parasites proliferate at a comparable rate to the wild-type (WT) and exhibit similar apoptotic responses. As expected, MIL-resistant parasites demonstrate decreased susceptibility to MIL, which reduces after the drug is withdrawn from culture. Our data demonstrate metacyclogenesis is elevated in MIL-resistant *L*. *major*, albeit these parasites display attenuated *in vitro* and *in vivo* virulence and standard survival rates in the natural sandfly vector, indicating that development of experimental resistance to miltefosine does not lead to an increased competitive fitness in *L*. *major*.

## Author Summary

Cutaneous Leishmaniasis (CL) is characterized by the appearance of ulcerative lesions on the skin, and results from infection with trypanosomatid parasites such as *Leishmania major*. Current treatments for CL are expensive and have a wide range of toxic side effects of variable severity. Miltefosine, a recently introduced treatment option, is the first oral drug for leishmaniasis treatment. Although widespread clinical resistance has not yet been established, miltefosine-resistant parasite populations are easily created in a laboratory environment. Through step-wise selection, we have created populations of *L*. *major* resistant to miltefosine. These resistant parasites grow at a similar rate to miltefosine-sensitive parasites and exhibit similar stress responses. Accordingly, miltefosine-resistant parasites display a decrease in tolerance when selective pressure of MIL is withdrawn from the population. There is no conferred resistance to treatment with other antileishmanial agents, though increased sensitivity to alternative treatments is observed in some instances. *Leishmania* undergoes a complex life cycle including the differentiation to highly infective forms, in a process termed metacyclogenesis. Experimental resistance to miltefosine increases metacyclogenesis in *L*. *major*, however resistant parasites display a lower fitness than their sensitive counterparts, as judged by their attenuated virulence *in vitro* and *in vivo*.

## Introduction

Leishmaniasis is caused by protozoan parasites of the genus *Leishmania*, and presents as a variety of clinical manifestations ranging from lesions on the skin to disseminated visceral infections [1]. Cutaneous leishmaniasis (CL) often results in self-resolving lesions, whereas visceral leishmaniasis (VL) is habitually fatal when left untreated. With an annual incidence of 2 million cases and a prevalence of more than 12 million, leishmaniasis is responsible for 70,000 deaths annually [2]. 88 countries have reported infection, resulting in 350 million individuals at risk for infection and an estimated 2.4 million disability-adjusted life years (DALYs) [2]. These statistics are grossly underestimated due to misdiagnosis and insufficient disease surveillance systems.

*Leishmania* species have a digenetic life cycle including both extracellular promastigote and obligate intracellular amastigote forms. Extracellular flagellated promastigotes reside in the midgut of the phlebotomine sandfly vector. Following infection in the mammalian host, promastigotes are engulfed by macrophages where they differentiate into non-motile amastigotes in the phagolysosome. This differentiation is triggered by environmental cues, mainly pH and temperature [3]. Current antileishmanial drugs include pentavalent antimony, amphotericin B, paromomycin, pentamidine, and miltefosine; most are toxic and expensive. To date, no successful vaccine exists, and the few antileishmanial drugs mentioned either risk becoming ineffective due to emerging resistance, or are limited in their use due to cost and parental administration [4, 5]. Miltefosine (MIL) is an alkylphosphocholine drug with demonstrated activity against various parasite species and cancer cells, as well some pathogenic bacteria and fungi [6]. Since its registration in 2002, miltefosine remains the only oral agent used for the treatment of all types of leishmaniasis. The U.S. Food and Drug Administration (FDA) recently (March 2014) approved Impavido (miltefosine) for the treatment of cutaneous, visceral and muco-cutaneous leishmaniasis. While the mechanism of action of MIL is not understood in its entirety, several studies have pointed at alterations in phospholipid metabolism, impairment of bioenergetic metabolism, and ultimately the induction of apoptosis as potential modes of actions [7–10]. Knowledge of experimental MIL resistance in *Leishmania* is limited to defects in drug internalization (defective inward translocation of MIL) and increased drug efflux [11]. Previous investigations in *L*. *donovani* have revealed the presence of several key point mutations in the P-type ATPase dubbed the LdMT (*L*. *donovani* miltefosine transporter) [12]. However, subsequent studies demonstrated that the LdMT alone was not sufficient to facilitate translocation, leading to the identification of the β-subunit LdRos3 and its importance to the function of the LdMT [13]. Mutations in the LdMT and Ros3 contribute to the MIL-resistant phenotype by significantly decreasing MIL uptake. Specifically, T420N and L856P mutations in the LdMT contributed to significantly decreased MIL uptake [12]. Other mutations identified in MIL-resistant *L*. *donovani* include W210 (LdMT) and M1 (LdRos3) [14]. Sequencing of the entire miltefosine transporter was performed in both *L*. *major* and *L*. *infantum*, and all identified sequence mutations differed from those previously detailed in *L*. *donovani* (L856P, T420N, W210, and M1) [15]. In the same study, no mutations were observed in the β-subunit Ros3 in any of the MIL-resistant populations. Widespread clinical resistance has not yet been demonstrated, nonetheless two *L*. *infantum* isolates from HIV co-infected patients have been reported to exhibit MIL resistance [16, 17]. The analysis of clinical isolates from patients infected with *L*. *donovani* that had relapsed to standard MIL therapeutic regimes demonstrated that the recovered parasites were significantly more tolerant to MIL [14]. None of the resistance markers i.e. point mutations aforementioned were found in the isolates. In the absence of a definitive mechanism of miltefosine resistance, the concept of fitness or “proficiency” of drug resistant pathogens is becoming more relevant and how the acquisition of resistance may impact the life cycle of the parasite, particularly its capacity to survive both in the insect and mammalian hosts and thus its ability to compete with wild type (sensitive) parasites [18–20]. Most of these studies are focused on antimony resistance in *L*. *donovani* and more recently, drug combinations [21]. Here we present the characterization and fitness of clonal lines of *L*. *major* that have experimentally acquired resistance to miltefosine, with relevance to survival in the mammalian host and phlebotomine vector.

## Materials and Methods

### Ethics statement

All studies using vertebrate animals were conducted in accordance with the U. S. Public Health Service Policy on Humane Care and Use of Laboratory Animals and followed the standards as described in the *Guide for the Care and Use of Laboratory Animals*. Per these standards, all vertebrate animal studies were conducted following review by the University of Notre Dame Institutional Animal Care and Use Committee under protocol #15–047 (approved October 16, 2012). The University of Notre Dame is credited through the Animal Welfare Assurance #A3093-01.

### Cell culture conditions

*Leishmania major* strain Friedlin V1 (MHOM/JL/80/Friedlin) promastigotes were cultured at 27°C in M199 medium (medium 199 (CellGro) supplemented with 10% heat-inactivated fetal bovine serum (FBS), 20 mM HEPES, 10 mM adenine, penicillin/streptomycin, hemin, biotin, L-glutamine, and 7.5% NaHCO_3_) and passaged every 3–4 days. Macrophages (RAW264.7 cell line) were cultured at 37°C with 5% CO_2_ in RPMI supplemented with 10% heat-inactivated FBS, penicillin/streptomycin, and L-glutamine, and passaged every 2–3 days.

### Generation of MIL-resistant populations

MIL-resistant cultures of *L*. *major* were generated using step-wise selection. Cultures were passaged every 3–4 days at an initial concentration of 5x10^5^ promastigotes/mL. Increasing concentrations of MIL (Sigma) were introduced to the cultures beginning with 2.5 μM MIL and successively to 5, 8, 10, 15, 20, 30, and 40 μM MIL. Cultures were exposed to an increased concentration of MIL when growth rates were equivalent to the growth rate of the wild-type (WT). To account for clonal variation, 2 clones of each resistant line were generating by plating in M199 plates as previously described [22]. Clones 1 and 2 were simultaneously maintained.

Growth rates were measured for each set of resistant populations and compared with the WT strain. Parasites were counted at an initial concentration of 5x10^5^ parasites/mL and growth was measured daily using a Neubauer chamber until the population reached stationary phase.

To further assess stability and fitness, two fluorescent FACS-based apoptotic markers were used to evaluate MIL-selection. Membrane permeability was assessed using the kit YO-PRO1 (Invitrogen) according to manufacturer’s recommendations. Briefly, samples were pelleted and washed in 1X M199 complete media. Following the wash, samples were resuspended in 1X M199 complete media and YO-PRO (Invitrogen) and Propidium Iodide (Invitrogen) were added and incubated for 20 minutes. Exposure of phosphatidylserine (PS) residues was investigated with Annexin-V-FITC (Miltenyi Biotec) following manufacturer’s instructions. Analyses were performed in a Beckman Coulter FC500 Flow Cytometer.

### Assessment of drug resistance

In order to assess the MIL-resistance achieved, the half-maximal effective concentration, EC_50_, was performed using the resazurin-based CellTiter-Blue (Promega) method as previously described [23]. Cultures were counted using a Neubauer chamber. 1x10^6^ parasites/mL were incubated for 48 hours at 27°C in M199 medium (CellGro) and appropriate concentrations of MIL (Sigma), pentamidine isethionate (Sigma), amphotericin B (Sigma), potassium antimony(III) tartrate hydrate (Sigma) and paromomycin sulfate salt (Sigma), were used in order to accurately evaluate the resistance. Solvent (DMSO) controls were used where appropriate. Hundred μL from each well were incubated at 37°C at 5% CO_2_ for 4 hours with 20 μL Cell Titer Blue (Promega). Fifty μL of 10% SDS were added to each well, and fluorescence was measured (555 nm λexc/580 nm λem) using a Typhoon FLA-9500 laser scanner (GE Healthcare) and analyzed with ImageQuant TL software (GE Healthcare). EC_50_ values were calculated by non-linear regression analysis using SigmaPlot (v 11.0). All experiments were done in triplicate with appropriate controls in each case.

### Partial sequencing of LmMT and LmRos3

Both WT and MIL-resistant cultures were sequenced for previously described point mutations in the *L*. *donovani* MT (T421N, L856P, W210*) and Ros3 subunit (M1) [14] and in *L*. *major* (G852D, M547del) [15]. DNA was amplified with primers outlined in S1 Table. PCR product sizes ranging from 149–277 bp were purified using the GeneJET Gel Extraction Kit (Thermo) and sent to the Genomics Core Facility at the University of Notre Dame for sequencing. Sequences were analyzed using ClustalX [24].

### RNA extraction and real-time PCR analysis

Total RNA was isolated from logarithmic and stationary phase promastigotes using Trizol Reagent (Invitrogen), reverse transcribed with SuperScript II Reverse Transcriptase (Invitrogen) after deoxyribonuclease I treatment with TURBO DNA-free Kit (Ambion, Invitrogen). All qRT-PCR reactions were performed in triplicate using SYBR Green (Invitrogen) fluorescence for quantification in a 7500 Fast Real-Time PCR System (Applied Biosystems). The ΔΔCƬ method was used to determine relative changes in gene expression [25] with data presented as fold change in the target gene expression in *L*. *major* MIL-resistant cultures normalized to internal control genes GAPDH and SOD, using *L*. *major* WT as a reference strain. Standard PCR conditions were: 95°C for 10 min, followed by 40 cycles of 94°C for 1 min, 60°C for 1 min, and 72°C for 2 min. Primer design was based on nucleotide sequences of *L*. *infantum* genes coding for the *L*. *donovani* MT, *L*. *donovani* Ros3, SHERP, GAPDH and SOD genes. All experiments were performed in triplicate with appropriate controls included in each case.

### Metacyclogenesis

Two different methods were utilized to assess metacyclogenesis as described previously [26]. Briefly, a Ficoll (Sigma) gradient was set-up using 4 mL of 20% Ficoll overlaid with 4 mL 10% Ficoll in M199 medium without FBS and 4 mL of 5-day stationary-phase culture in M199 medium laid on top. The step gradients were centrifuged at room temperature for 10 min at 1300 x g without braking or acceleration to separate out the layers. The top two layers of the gradient were recovered and the percentage of metacyclic parasites was determined by counting in a Neubauer chamber before and after the enrichment procedure. For agglutination analysis, 5-day stationary-phase cultures were pelleted and resuspended in 1 mL M199 medium (CellGro) and 10 μL peanut agglutinin (50 μg/mL) (Sigma) was added. After 30 minutes of room temperature incubation, samples were centrifuged at 200g for 10 minutes. The supernatant was recovered and the percentage of metacyclic parasites was determined by counting in a Neubauer chamber before and after the enrichment procedure. All experiments were done in triplicate.

### Macrophage infections

RAW264.7 murine macrophage cells were counted using Trypan Blue (Amresco) and plated at 5x10^5^ cells/well in 12-well plates. Infections were performed with metacyclic parasites isolated as described above. Infections were carried out at a multiplicity of infection (MOI) of 10 parasites per macrophage. Free parasites were removed by one wash with RPMI without FCS 6 h post-infection and samples collected at 6, 12, 24 and 48 h post-infection by DiffQuick staining of cytospin whole-cell preparations and visualized with light microscopy. All infections were done in triplicate and at least two independent experiments were performed.

### Sandfly infections

*Phlebotomus papatasi* (Origin: Turkey, PPTK) was reared in the Department of Biological Sciences, University of Notre Dame, according to conditions previously described [27]. For the experiment, three-to-five day old female sandflies were used. Two groups, one experimental and one control, each containing 50 female and 10 male sandflies were placed in a 500 mL plastic container (ø = 6.3 cm, height = 6.5 cm) (Thermo-Nalgene) covered with a piece of nylon mesh (0.5mm). Blood feeding was performed through a young chicken skin membrane attached to a feeding device. Prior to sandfly feeding, fresh mouse blood was heat inactivated for 30 min at 56°C. Infection of sandflies with *L*. *major* FVI strain promastigotes was done by addition of 1×10^7^ logarithmic parasites/mL into the blood meal. Sixteen to twenty four hours after blood feeding, the presence or absence of blood in the sandfly digestive tract was verified by anesthetizing flies with CO_2_ and observing the midgut distension under a stereomicroscope (Carl Zeiss). One week post-blood meal, midguts of blood-fed sandflies were individually dissected and thoroughly homogenized in 30 μl PBS buffer (pH 7.4) using a hand held tissue homogenizer and pestle. Parasites were counted in a Neubauer chamber.

### Mouse strains and infections

5x10^5^ metacyclic parasites isolated by peanut agglutinin (see above) from stationary cultures of *L*. *major* FVI were injected subcutaneously in the left hind footpad of Balb/c mice, as previously described [26]. Lesion development was monitored by measuring weekly the thickness of the footpad using a Vernier caliper. Number of parasites at lesion site were enumerated by limiting dilution assay [28]. Cell lines were passaged at least once through mice before performing *in vivo* virulence studies to minimize the loss of virulence after prolonged in vitro culture.

### Statistics

Significance was determined by p-values calculated from a two-tailed student’s T-test in GraphPad Prism 6.0 unless otherwise stated.

### Accession numbers

*L*. *donovani* MT: GenBank accession number AY321397.1; *L*. *donovani* Ros3 GenBank accession number DQ205096.1; SHERP: GenBank accession number XM_001683391; GAPDH: GenBank accession number XP_001684904, and SOD: GenBank accession number XP_001685502.

## Results and Discussion

### Selection of MIL-resistant populations of *L*. *major*

**Fig 1:**
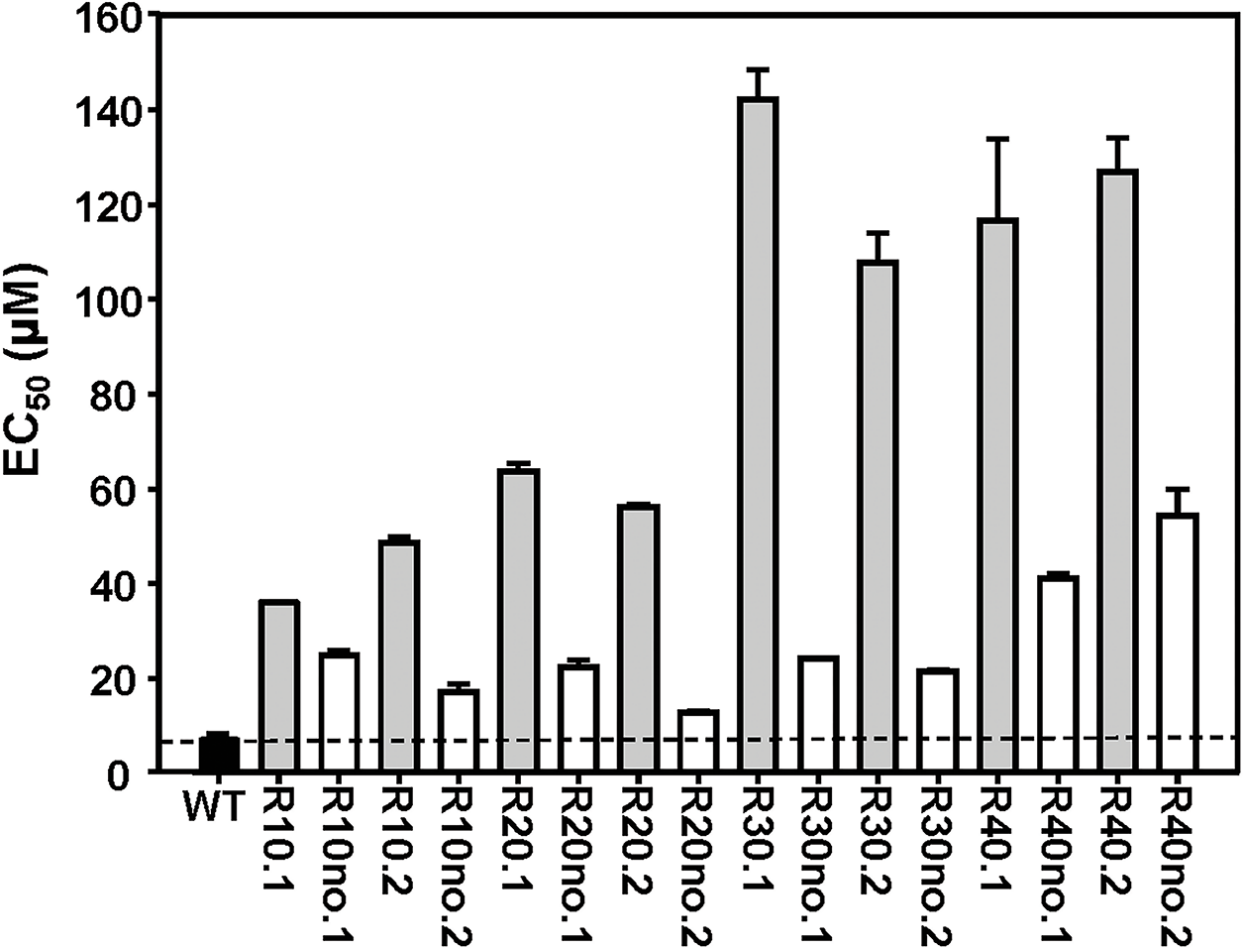
Susceptibility of MIL-resistant *L*. *major* FVI populations generated by step-wise selection and determined by EC_50_ analysis. 1×10^6^ Log-phase parasites were incubated in the presence of a range of drug concentrations for 48 hours at 27°C, and the surviving cells were quantified with Cell Titer Blue proliferation assay using a Typhoon FLA-9500 laser scanner. Populations of parasites were grown in increasing concentrations of MIL ranging from 10 μM (R10) to 40 μM (R40), showing increased resistance to MIL. Horizontal dashed line represents WT threshold for MIL resistance. “Rno” are resistant lines grown in the absence of MIL for at least 75 passages. Results are the average of triplicate experiments ± SD.

*L*. *major* FVI MIL-resistant parasites were generated using step-wise selection up to 40 μM MIL. Parasites were unable to proliferate in higher MIL concentrations, likely due to reaching the critical micellar concentration of MIL leading to degradation of the membrane due to the detergent effects of MIL [29]. FVI WT promastigotes were plated in solid M199 media and two random clones were used for MIL selection in flasks. In order to assess the degree of MIL-resistance in our lab populations of *L*. *major* we measured EC_50_ values using the resazurin-based CellTiter-Blue (Promega) assay. MIL-resistant cultures exposed to the highest concentrations of MIL (30 μM, 40 μM), and labeled R30 and R40 herein, have accordingly higher EC_50_ values than R10 and R20 (Fig 1). MIL-resistant cultures growing in the absence of MIL exhibited lower EC_50_ values than their counterparts under constant MIL-selection. However, it is important to note that this decreased EC_50_ value of MIL-resistant *L*. *major* is still higher than the EC_50_ of WT *L*. *major* cultures (Fig 1, dotted line) after at least 95 passages (2 passages per week, *ca* 11 months). This suggests that once any degree of resistance is accrued MIL-resistant cultures do not revert back to WT phenotype, despite the removal of MIL selective pressure (Fig 1). It is worth noting that a different resistant phenotype may be obtained if drug selection is performed in axenic promastigotes or intracellular amastigotes, as shown for paromomycin selection in antimony-resistant *L*. *donovani* [17, 30].

### Phenotypic characterization

**Fig 2:**
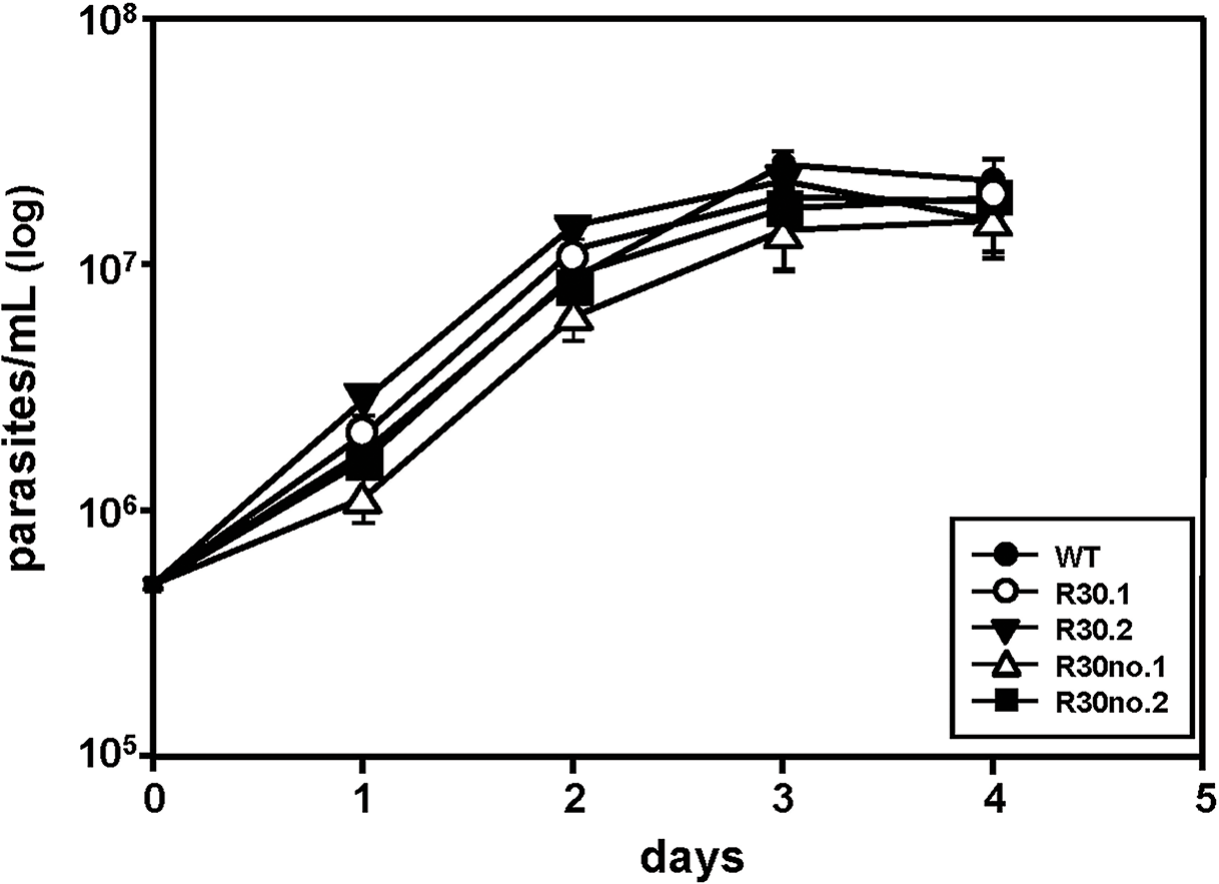
Growth curves of *L*. *major* WT and MIL-resistant promastigotes growing in the presence of 30μM MIL or absence of MIL selection. Log-phase promastigotes cultures were counted daily until they reached stationary phase. Concentration was determined microscopically by counting in a Neubauer chamber. Results are the average of triplicate experiments ± SD.

We next determined any difference in growth patterns between the sensitive (WT), resistant (R30) and resistant grown in the absence of MIL (R30no) *L*. *major* populations. Growth curves showed that MIL-resistant *L*. *major* proliferation is similar to *L*. *major* WT and cured lines (Fig 2), indicating that increased MIL exposure has no effect on proliferation in *L*. *major*. We used a FACS-based approach to detect two different apoptotic markers i) membrane permeability and ii) PS exposure to determine the response of parasite to stress after MIL selection. *L*. *major* R30 cell lines exhibit minimal stress and are comparable to WT populations judging the histogram levels corresponding to Annexin V and YO-PRO as analyzed by flow cytometry (S1 Fig).

Experimental MIL-resistance in *L*. *donovani* has previously been attributed to identified point mutations in the MT and Ros3 subunit (T421N, L856P, W210, and M1) [31]. We sequenced the regions of the transporter and subunit in two independent clones of the R40 line (highest concentration; R40.1 and R40.2) that had been under drug selection for at least 75 passages. As shown in Table 1, these mutations were not found in our lab populations. These results are in accordance with previous characterization of MT in MIL-resistant *L*. *major* [15]. Two genuine mutations identified in the *L*. *major* MT were pinpointed for this study: a three-nucleotide deletion (M547del) and a transition mutation (G852D) [15]. As seen in Table 1, our lab populations displayed identical sequences to WT. Although our data do not eliminate the possibility of other unidentified genetic mutations having a role in MIL-resistance in *L*. *major*, it is interesting to observe that even at higher concentrations (R40) and after long-term exposure to MIL (at least 75 passages) none of the reported mutations were found.

**Table 1:** Identification of point mutations previously identified in MIL-resistant *L*. *donovani* (T421N, L856P, W210*, M1) and *L*. *major* (G582D, M547del).

**T421N** *		
*L*. *major* wild-type	1256	TCGAA**G**CCGAC
*L*. *major* R40 clone 1	1256	TCGAC**G**CCGAC
*L*. *major* R40 clone 2	1256	TCGAC**G**CCGAC
**L856P** *		
*L*. *major* wild-type	2562	GGTGG**T**GCAGT
*L*. *major* R40 clone 1	2562	GTCGG**T**GGTGC
*L*. *major* R40 clone 2	2562	GTCGG**T**GGTGC
W210* *		
*L*. *major* wild-type	625	CACGA**G**CAAGC
*L*. *major* R40 clone 1	625	CACGA**G**CAAGC
*L*. *major* R40 clone 2	625	CACGA**G**CAAGC
M1* *		
*L*. *major* wild-type	1	AT**G**CTCGG
*L*. *major* R40 clone 1	1	AT**G**CTCGG
*L*. *major* R40 clone 2	1	AT**G**CTCGG
**G852D** *		
*L*. *major* wild-type	2518	GCCAG**C**TGCAT
*L*. *major* R40 clone 1	2518	GCCAG**C**TGCAT
*L*. *major* R40 clone 2	2518	GCCAG**C**TGCAT
**M547del** * * *		
*L*. *major* wild-type	1635	CCATC**TGA**ATCCA
*L*. *major* R40 clone 1	1635	CCATC**TGA**ATCCA
*L*. *major* R40 clone 2	1635	CCATC**TGA**ATCCA

Previously identified mutations were sequenced and are indicated with an asterisk (*) and highlighted in bold font, using *L*. *major* FVI wild-type as the reference strain. No mutations were detected in any of the resistant lines.

### Cross-resistance of MIL-resistant L. major populations to other antileishmanials

We investigated the possibility of any conferred resistance to alternative antileishmanial treatments by measuring EC_50_ values as described in Material and Methods. No cross-resistance was found in any of the R30 clones or cured lines to amphotericin B, antimony (III) and paromomycin (Table 2). Interestingly, miltefosine resistance significantly increases the sensitivity of the parasite to treatment with pentamidine 3-fold lower than WT (Table 2). When MIL has been withdrawn, the sensitivity of the parasite to this particular treatment is restored to levels comparable with the wild-type (Table 2), suggesting a potential synergistic mechanism. A similar synergy has been reported for sitamaquine/pentamidine combinations in *L*. *donovani* [32], although the use of a combined therapy of miltefosine and pentamidine is hindered by the high toxicity of pentamidine [33]. Lastly, treatment of R30 MIL-resistant cultures with paromomycin had a significant effect on the sensitivity (ranging from 2–4 fold lower than WT) of one of the clones (R30.2), indicative of potential clonal variability.

**Table 2:** MIL resistance in *L*. *major* FVI promastigotes does not confer cross-resistance to alternative antileishmanials.

Drug	LmWT	LmR30.1	LmR30no.1	LmR30.2	LmR30no.2
**Miltefosine (μM)**	6.68±0.43	142.29±6.24	24.08±0.17	107.80±6.16	21.39±0.43
**Amphotericin B (nM)**		**−**	**−**	**−**	**−**
	52.36±11.37	69.61±15.05	44.38±4.03	46.36±0.57	64.13±14.41
**Antimony (III) (μM)**		**s**	**−**	**−**	**−**
	11.66±0.08	5.43±0.70	10.04±0.98	9.43±0.99	14.77±2.22
**Pentamidine (μg/ml)**		**s**	**−**	**s**	**−**
	3.23±0.43	0.93±0.10	3.13±0.35	1.71±0.12	3.57±0.34
**Paromomycin (μM)**		**s**	**s**	**s**	**s**
	161.91±9.78	41.56±6.52	108.56±3.02	99.54±6.82	94.05±3.00

1x10^6^ parasites/mL were incubated in increasing concentrations of amphotericin B (nM), pentamidine (μg/mL), paromomycin (μM), or antimony (III) (μM) for 48 hours at 27°C, using solvent controls where appropriate. Surviving cells were determined through the proliferation Cell Titer Blue assay using a Typhoon FLA-9500 laser scanner. Results are the average of triplicate experiments ± SD. (−) indicates no cross-resistance demonstrated as compared to WT, and (s) indicates an increased susceptibility to treatment.

### Metacyclogenesis in MIL-resistant parasites

**Fig 3:**
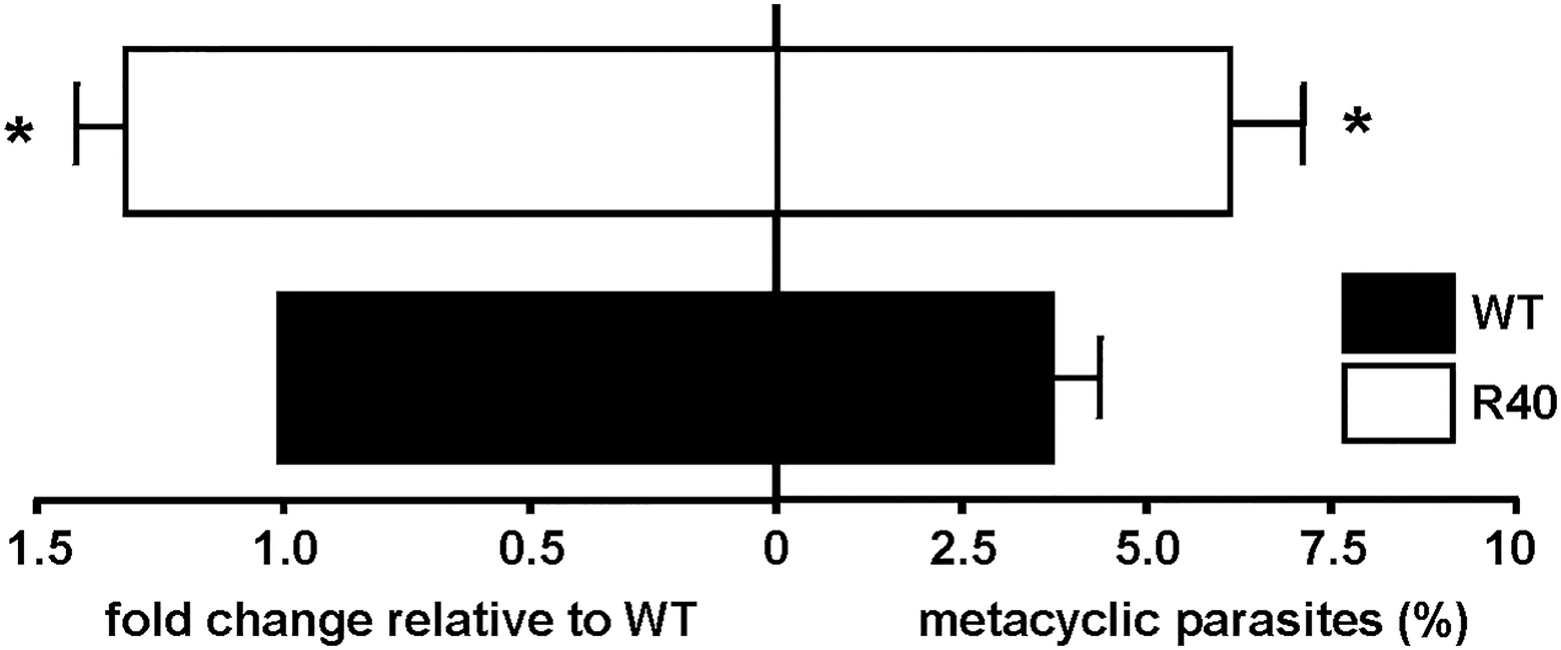
Metacyclogenesis in WT and MIL-resistant *L*. *major*. *L*. *major* promastigotes resistant to MIL exhibit increased metacyclogenesis as determined by qRT-PCR of SHERP expression relative to housekeeping gene GAPDH and normalized to WT expression levels (left). 5-day stationary parasites were subjected to peanut agglutination and Ficoll-400 gradients and percentage of metacyclics is shown (right). Results are the average of triplicate experiments ± SD. Statistical differences determined with a Student’s *t* test relative to control values (* *p*<0.05)

Procyclic *L*. *major* promastigotes differentiate into highly virulent metacyclic promastigotes during metacyclogenesis [34]. This process occurs in the midgut of sandflies and can be mimicked *in vitro* when acidification occurs in the medium. Due to the lack of phenotypic differences in our clonal lines we performed the following *in vitro* and *in vivo* experiments with the R40.2 line. We enriched metacyclic promastigotes by Ficoll 400 step gradient and peanut agglutination, as described in Material and Methods. Analyses of metacyclogenesis showed that *L*. *major* R40 had higher percentages (2-fold) of metacyclics than *L*. *major* WT (Fig 3, right panel). qRT-PCR was used to amplify SHERP gene, which is almost exclusively and highly expressed in infective and non-replicative stages of the parasite [35]. SHERP expression was significantly elevated in R40 parasites (Fig 3, left panel), confirming our metacyclic enrichment approaches. Increased metacyclogenesis has been reported in antimony-resistant *L*. *donovani* clinical isolates [36], and metacyclogenesis is regarded as a major contributor to the fitness of the parasite. In New World cutaneous species, *L*. *mexicana* resistant to Glibenclamide, an ATP-binding-cassette (ABC)-transporter blocker exhibited a reduced expression of the Meta-1 protein [37].

### *In vitro* and *in vivo* infection studies

**Fig 4:**
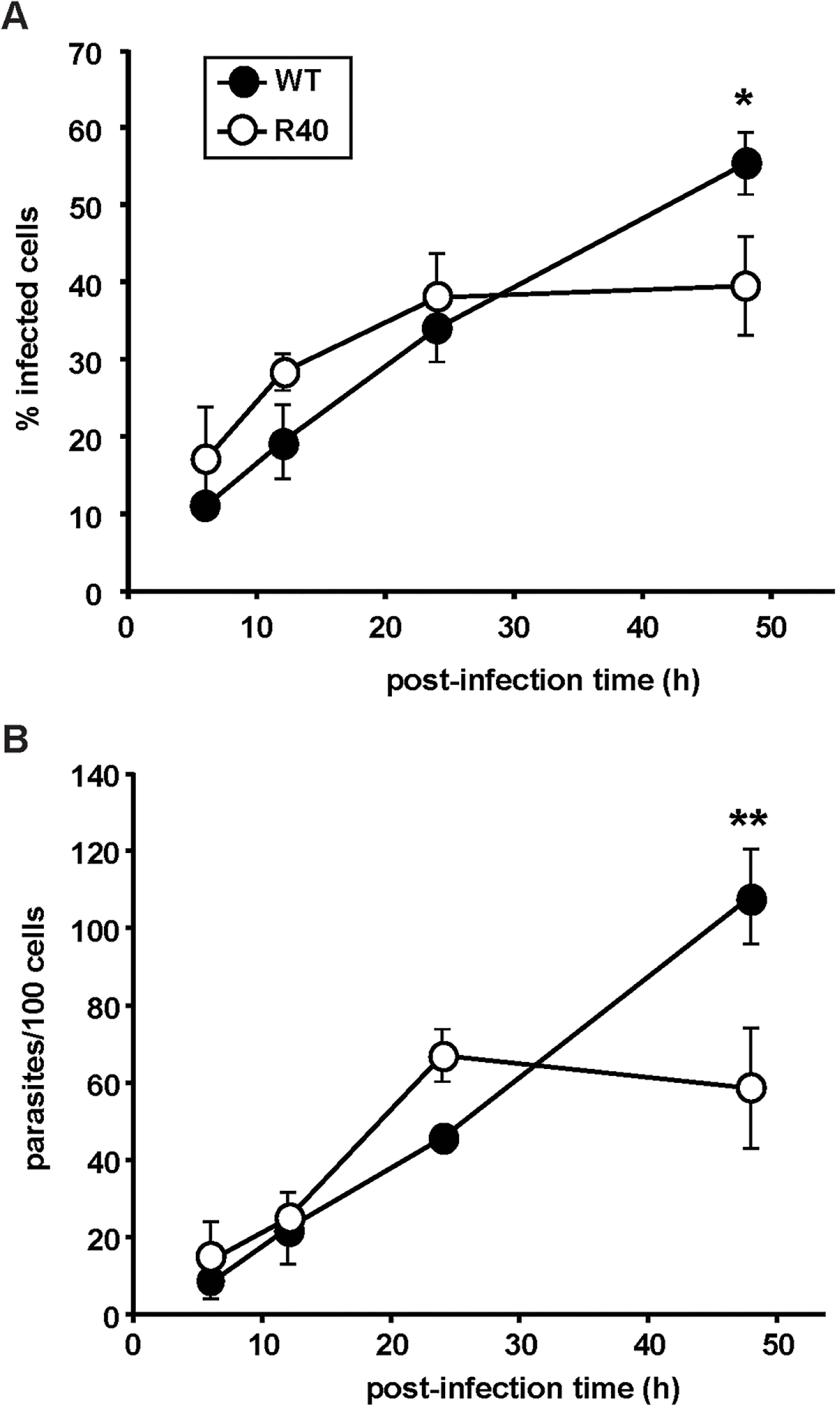
Host cell infection assay. Early stages of macrophage invasion are similar between *L*. *major* WT and R40, as determined by infection of RAW264.7 murine macrophages. Metacyclic parasites were incubated in the presence of macrophages at a MOI of 10 metacyclic parasites per macrophage and cells were collected at 6h, 12h, 24h, and 48h. Samples were stained and infection was determined through light microscopy. **(A)** The percentage of infected macrophages, and **(B)** the number of parasites/100 cells were recorded. Results are the average of triplicate experiments ± SD. Statistical differences determined with a Students *t* test relative to control values (* *p*<0.05; ** *p*<0.01)

The stationary phase-specific differences of R40 primed us to study their capacity to infect RAW264.7 murine macrophage cells. We routinely passage our *L*. *major* cell lines through Balb/c mice to compensate for the loss of virulence due to *in vitro* culture. 5-day stationary cultures were subjected to peanut agglutination, and R40 and WT lines were incubated with RAW264.7 cells at a multiplicity of infection of 10 metacyclics per host cell. Intracellular parasite burden was determined by nuclear staining and microscopy at 6, 12, 24, and 48 h postinfection. Initial levels of R40 infections are comparable to the control (Fig 4A). A significant difference in R40 infectivity was apparent 48 hours post infection. This was further corroborated by decreased intracellular proliferation of R40 cells 48 hours post infection by over 20% (Fig 4B). Pentamidine-resistant *L*. *mexicana* showed no differences in the *in vitro* infectivity in resident mouse macrophages when compared with the wild-type clone [38].

In contrast, higher metacyclogenesis levels in clinical isolates of *L*. *donovani* resistant to antimony translated into higher *in vitro* infection levels [36].

**Fig 5:**
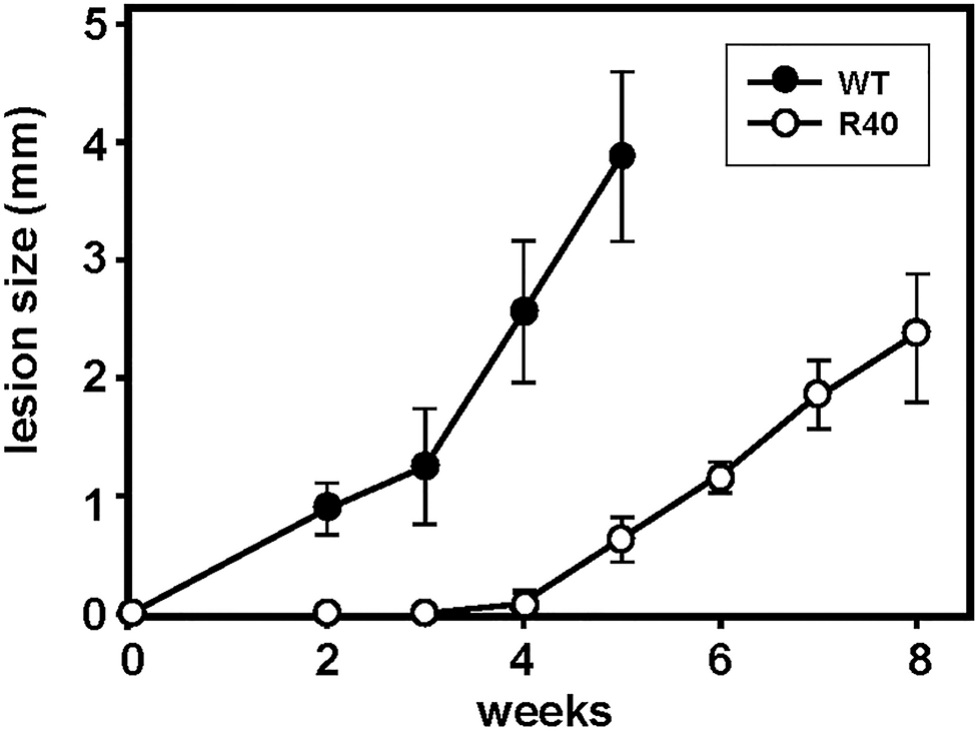
Virulence of WT and MIL-resistant *L*. *major*. R40 demonstrate attenuated virulence *in vivo* compared with WT promastigotes. 1×10^6^ WT (n = 5) and R40 (n = 6) metacyclic promastigotes were injected into the footpads of female BALB/c mice. Lesion size was recorded weekly by taking measurements of footpad thickness with a Vernier caliper, results are averages ± SD.

We next investigated the virulence of WT and R40 using an established experimental mouse infection [39]. Control and R40 were normalized for virulence through one passage in Balb/c mice [40]. 10^5^ WT and R40 metacyclic parasites were inoculated into the hind footpad of groups of five-six female Balb/c mice. A Vernier caliper was used to monitor lesion formation by measuring the increase in footpad size weekly. Control parasites attained a lesion size of ca. 4 mm, 5 weeks after inoculation and resulted in necrotic lesions (Fig 5). Interestingly, R40 were highly attenuated and lesions were only apparent 4 weeks after infection. Our observations *in vitro* with R40 cells showing a decreased infectivity and intracellular proliferation seem to have extended well to an *in vivo* mouse model. Amphotericin-resistant *L*. *mexicana* parasites were able to infect Balb/c mice, but the resulting lesion growth was slower than that after infection with susceptible parasites [41]. In contrast, several clinical isolates of *L*. *donovani* resistant to pentavalent antimonials showed a greater virulence in a mouse model of visceral leishmaniasis [42]. Importantly, our data suggest that metacyclogenesis alone is not a reliable marker of fitness, at least in MIL-resistant *L*. *major*, and *in vitro* and *in vivo* studies are necessary to further assess its competitive fitness. In this scenario, the *L*. *major /* MIL combination resembles the reduction in fitness widely observed in *Plasmodium falciparum* populations resistant to chloroquine [43].

### Sandfly infection studies

**Fig 6:**
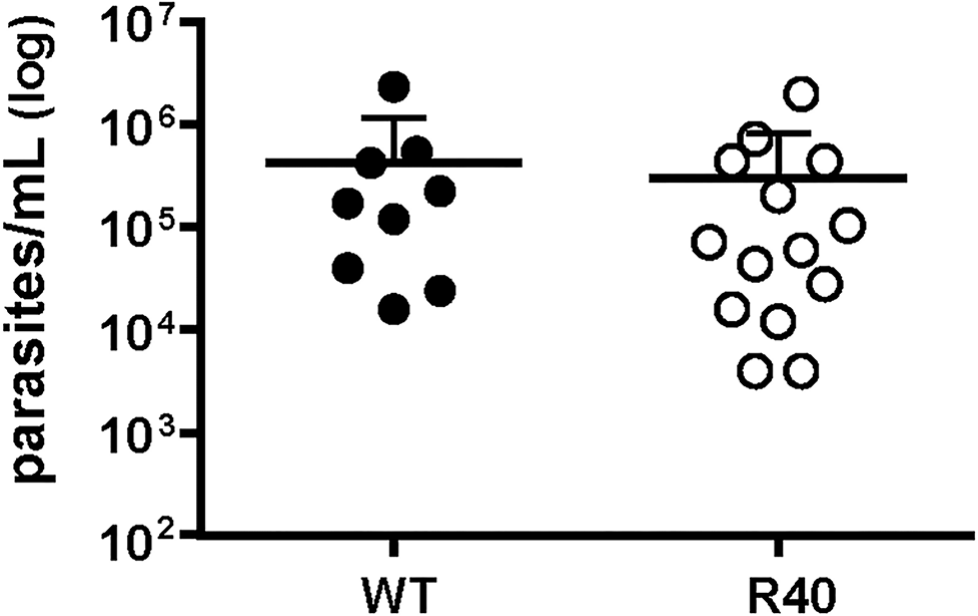
WT and R40 *L*. *major* parasites exhibit comparable ability to colonize and survive in the sandfly vector. *P*. *papatasi* were fed with heat-inactivated fresh mouse blood mixed with 1×10^7^ parasites/mL of both *L*. *major* WT and R40. Blood-fed sandflies (WT: n = 9, R40: n = 14) were maintained for one week on a sucrose diet, after which the midgut was dissected. Midguts were placed in 50 μL 1X PBS and crushed with a pestle. Parasite presence in each midgut was assessed by counting with a Neubauer chamber. No significant differences were observed.

Fitness of *Leishmania* parasites is linked to transmission success in the natural insect vector, therefore we tested whether MIL resistance would impact the capacity of *Leishmania* to survive in the natural sandfly vector. Three-to-five day old female *Phlebotomus papatasi* (Origin: Turkey, PPTK) sandflies were infected with 1×10^7^ logarithmic parasites/mL as described in Material and Methods. 24h post-blood meal, the presence or absence of blood in the sandfly digestive tract was verified and one week post-blood meal, 9 midguts of blood-fed sandflies infected with WT and 14 midguts from the R40 group were individually dissected. Parasite load per individual midgut was assessed. No significant differences were observed between the two groups (Fig 6) suggesting that MIL resistance does not affect the survival capacity of *L*. *major* in the natural vector.

In summary, as shown for *L*. *donovani* [44], the generation of experimental resistance to MIL is easily achieved by step-wise selection in *L*. *major*. Axenic resistant promastigotes proliferate as control cells, and the phenotype is stable. As suggested by our data, metacyclogenesis is an important process in the life cycle of the parasite, but should be carefully interpreted as a fitness marker. A combination of *in vitro*, *in vivo* and vector studies are necessary to fully assess the competitive fitness of MIL-resistant *L*. *major*, and studies would be further strengthened with the use of recent clinical isolates of both MIL-sensitive and MIL-resistant *L*. *major* parasites. Further studies will attempt to understand the impaired ability of MIL-resistant *L*. *major* to survive in the mammalian host at the molecular level. Overall, our findings are relevant for current and future antileishmanial chemotherapy strategies.

## Supporting Information

S1 Fig Flow cytometry analysis of MIL-resistant *L*. *major* promastigotes. WT, *L*. *major* FVI promastigotes grown in 40μM MIL, and R40 promastigotes where the MIL selection has been withdrawn, using two different apoptotic markers **(A)** Annexin V and **(B)** YO-PRO.
(TIF)

S1 Table List of primers used in this study. (DOCX)
