# Supplementary figures and images for: Fitness and Phenotypic Characterization of Miltefosine-Resistant Leishmania major
Source: PLoS Negl Trop Dis. 2015 Jul 31;9(7):e0003948. doi: 10.1371/journal.pntd.0003948 (PMC4521777; doi:10.1371/journal.pntd.0003948)

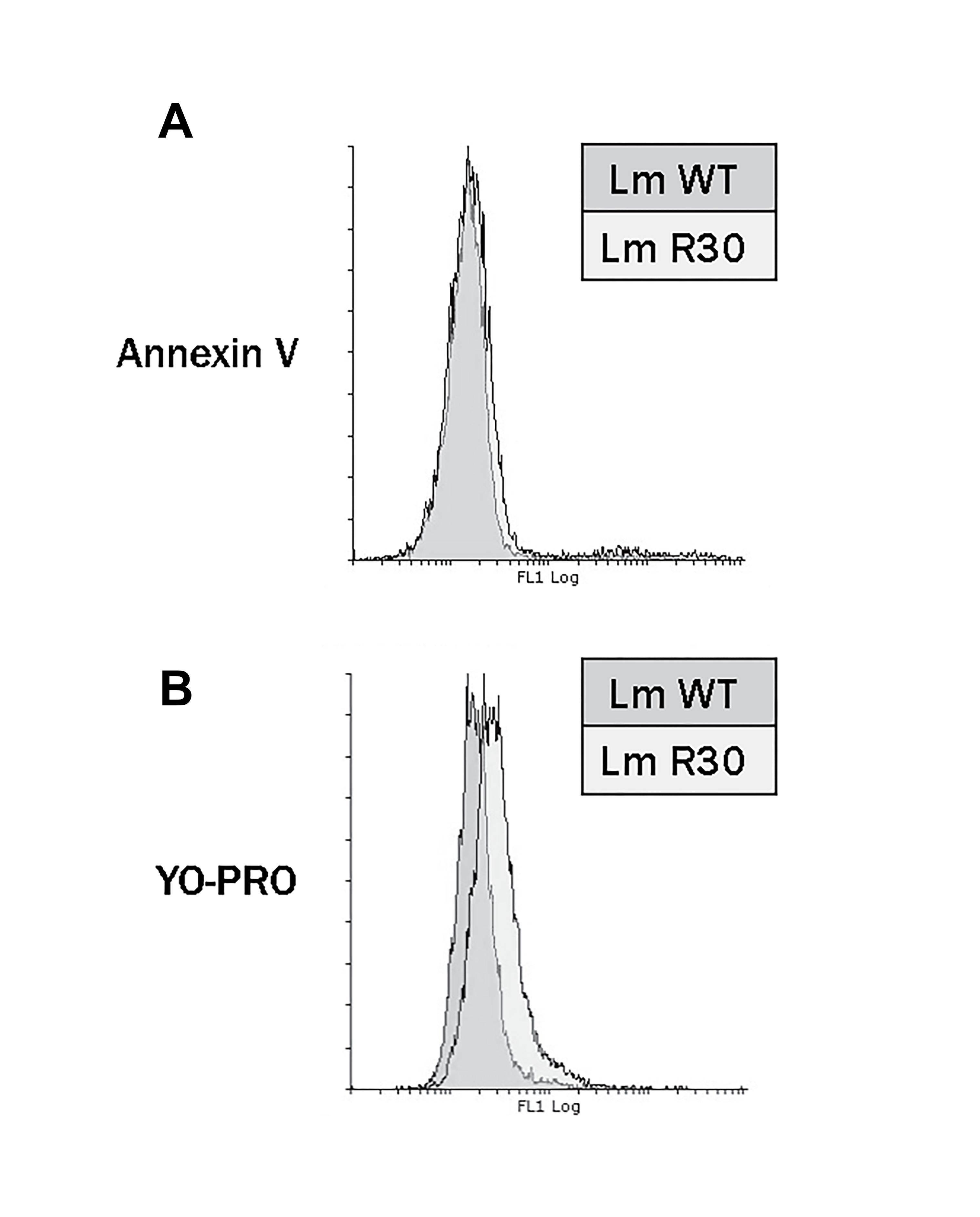

Supplement: S1 Fig — WT, L. major FVI promastigotes grown in 40μM MIL, and R40 promastigotes where the MIL selection has been withdrawn, using two different apoptotic markers (A) Annexin V and (B) YO-PRO. (TIF) [file pntd.0003948.s001.tif]
