# Supplementary material for: Fitness and Phenotypic Characterization of Miltefosine-Resistant Leishmania major
Source: PLoS Negl Trop Dis. 2015 Jul 31;9(7):e0003948. doi: 10.1371/journal.pntd.0003948 (PMC4521777; doi:10.1371/journal.pntd.0003948)
Supplement: S1 Table — (DOCX) [file pntd.0003948.s002.docx]

| **Primer** | **Sequence 5’-3’** |
| --- | --- |
| Ld W210* | Fwd: CGA GGA AGG ACA GGC ATT TA  Rev: GGT CTG GCT TGC TCG TGT C |
| Ld T421N | Fwd: CTG CCT ATG ATG GAG TAC A  Rev: GCC TAG CCC CTT CGA CTC |
| Ld L856P | Fwd: CCA ACG ACG TGT CCA TGA T  Rev: AAG GTC AGC ATC CAT CCA TC |
| Ld M1* | Fwd: TAC AGC TTT TGC TGC CCT TT  Rev: ATA GCA GCG ACT GCC AGA AT |
| Ld SHERP | Fwd: CGA CAA GAT CCA GGA GCT GAA GGA C  Rev: CCT TGA TGC TCT CAA CCG TGC TG |
| Lm MT | Fwd1: CCC TTT CGT TGT TCA CAG AAA CGC GCG GG  Fwd2: GGA CAT CCA CCC CGG TGA CG  Fwd3: GGA GTA CAT GAA CAA CCG CTG GCG GC  Fwd4: GCC GGC AAG TCC CTG CAC AAC CGC  Fwd5: CGC TCT TCC GCA ACG CCA GCT GC  Rev: CGC TCT TCC CTC GCC AAG TGA CTA CC |
| Lm Ros3 | Fwd1: GCA CTA CTC TCA ACC TCG TGT TTG CG  Fwd2: CGG CCA ACG GAA CGA GTC TGG CGC C  Rev: GGT AAA ACT GCT CTA TTG ATG ATG GC |
